# Supplementary material for: Directionality bias underpins divergent spatiotemporal progression of Alzheimer-related tauopathy in mouse models
Source: bioRxiv. 2024 Jul 23:2024.06.04.597478. Originally published 2024 Jun 6. Preprint. [Version 2] doi: 10.1101/2024.06.04.597478 (PMC11185722; doi:10.1101/2024.06.04.597478)
Supplement: Supplement 1 [file media-1.pdf]

Supplement to "Directionality bias underpins divergent spatiotemporal progression of Alzheimer-related tauopathy in mouse models"

Justin Torok[1], Christopher Mezias[2], and Ashish Raj[1,*]

[1]University of California, San Francisco, Department of Radiology, San Francisco, CA, 94143, United States
[2]Cold Spring Harbor Laboratory, Department of Neuroscience
[*]Address correspondence to ashish.raj@ucsf.edu

Supplemental Figures and Tables

Name	Mouse Model Description	$R_{\text{fit-s}}$	s_{opt}
**IbaStrInj** [1]	PS19 mice seeded with synthetic PFFs from 2N4R P301S tau (T40/PS) and from truncated P301L tau (K18/PL) in the right CP and right MOp	**0.56*****	0.78
**IbaHippInj** [1]	PS19 mice seeded with synthetic PFFs from 2N4R P301S tau (T40/PS) and from truncated P301L tau (K18/PL) in the right DG	**0.59*****	0.73
**Hurtado** [2]	Unseeded doubly transgenic PS19/PDAPP mouse	**0.78*****	0.65
**DS4** [3]	PS19 mice seeded with tau from AD brain homogenate in the left CA1 area	**0.70*****	0.50
**DS6** [3]	PS19 mice seeded with P301S mouse-derived, fibril-like cytoplasmic inclusions (“threads”) in the left CA1 area	**0.76*****	0.56
**DS6 110** [3]	PS19 mice seeded with a 1:10 dilution of the **DS6** tau strain in the left CA1 area	**0.56*****	0.53
**DS7** [3]	PS19 mice seeded with recombinant fibrils with prominent nuclear inclusions (“speckles”) in the left CA1 area	**0.75*****	0.54
**DS9** [3]	PS19 mice seeded with recombinant fibrils with prominent nuclear inclusions (“speckles”) in the left CA1 area	**0.67*****	0.64
**DS9 110** [3]	PS19 mice seeded with a 1:10 dilution of the **DS9** tau strain in the left CA1 area	**0.69*****	0.51
**BoludaDSAD** [4]	PS19 mice injected with DSAD brain homogenate in the left CA1 and left primary somatosensory areas (LH)	**0.43*****	0.78
**BoludaCBD** [4]	PS19 mice injected with CBD brain homogenate in the left CA1 and left primary somatosensory areas (LH)	**0.57*****	0.45

Table S1: Summary of key NexIS:directed results. List of the tauopathy datasets explored here with descriptions of each experiment’s mouse genetic background, injection site, and type of tau injected. The Pearson’s correlation values for the directionality-fitted models ($R_{\text{fit-s}}$) and the optimal directionality parameter (s_{opt}) values are provided. All studies were quantified tau pathology within hemispheres ipsilateral and contralateral to the injection site separately with the exception of Hurtado, which was bilaterally averaged. PFF – preformed fibrils; DSAD – Down Syndrome Alzheimer’s disease; CBD – corticobasal dengeneration. * – $p < 0.05$; ** – $p < 0.01$; *** – $p < 0.001$.

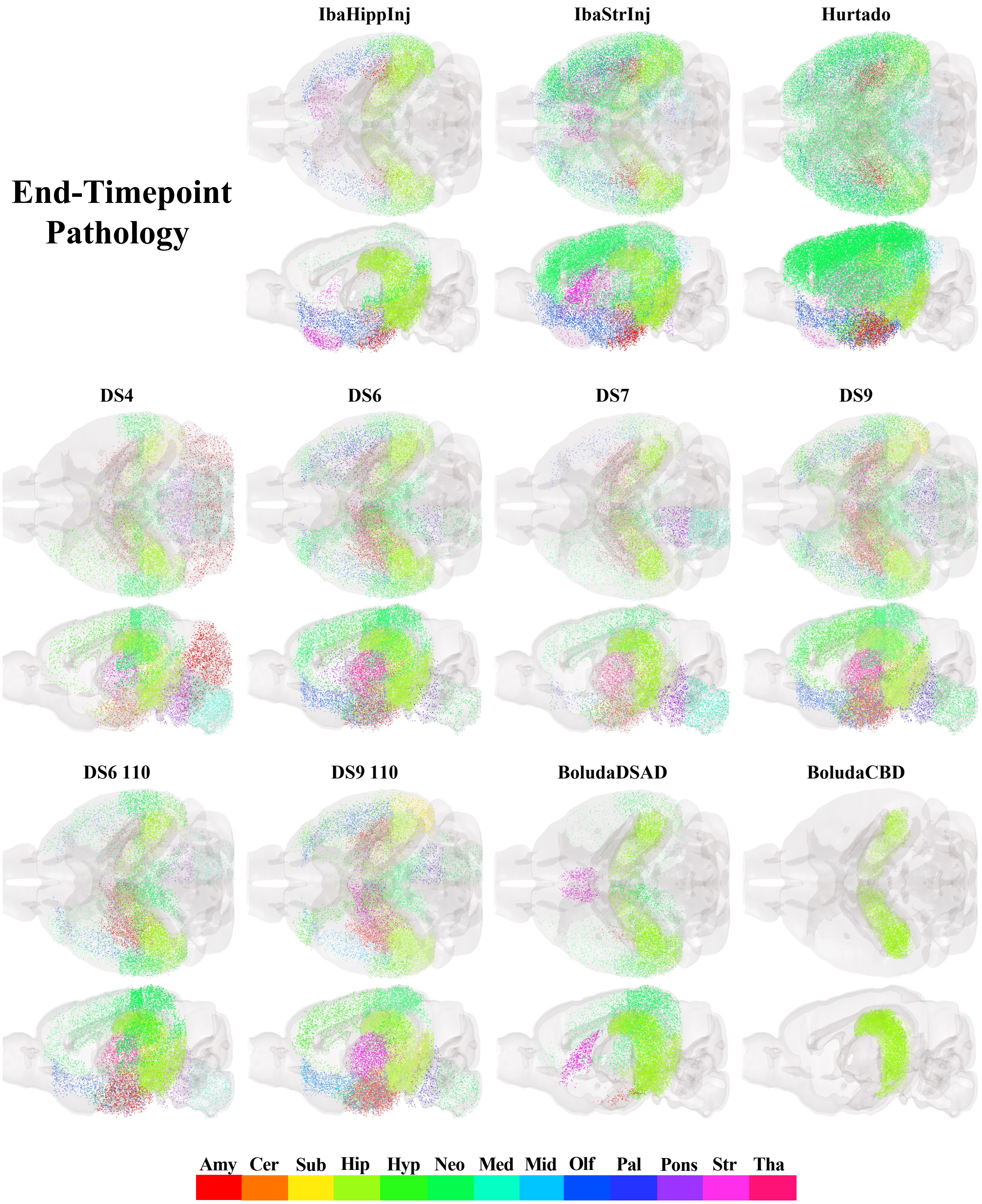

Figure S1: End-timepoint pathology glass brains. End-timepoint pathology for each of the twelve mouse tauopathy datasets, plotted in axial and sagittal views. See **Table 1** for descriptions of these datasets.

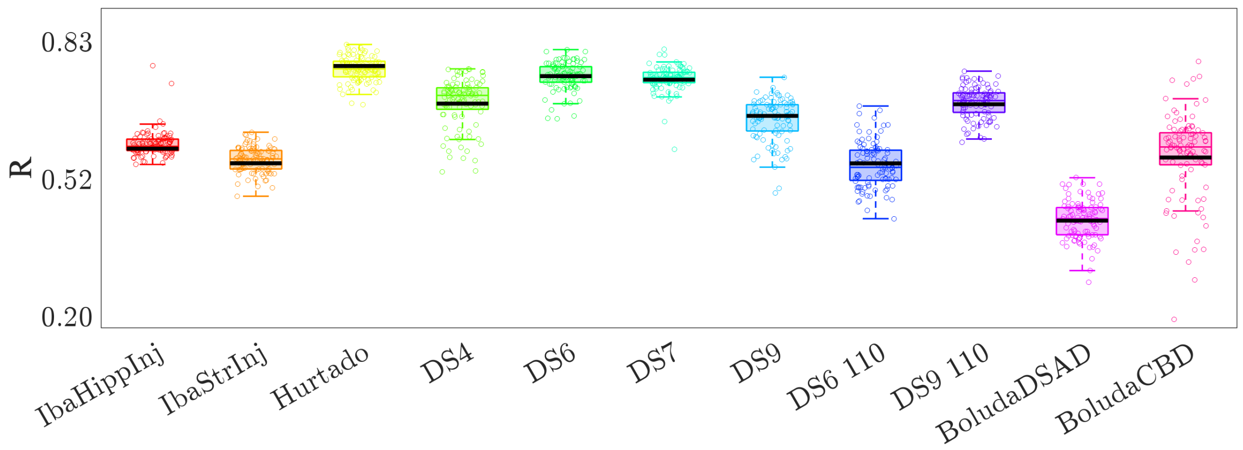

Figure S2: Bootstrapping analysis reveals no fit bias Boxplots and scatter representing the Pearson's R values obtained when NexIS:fit-s was fit on 100 random subsets of 80% of brain regions, alongside the fits to all regions (black lines). The fitting to all regions does not appear to introduce strong bias. See **Table 1** for descriptions of these datasets and **Methods** for more details.

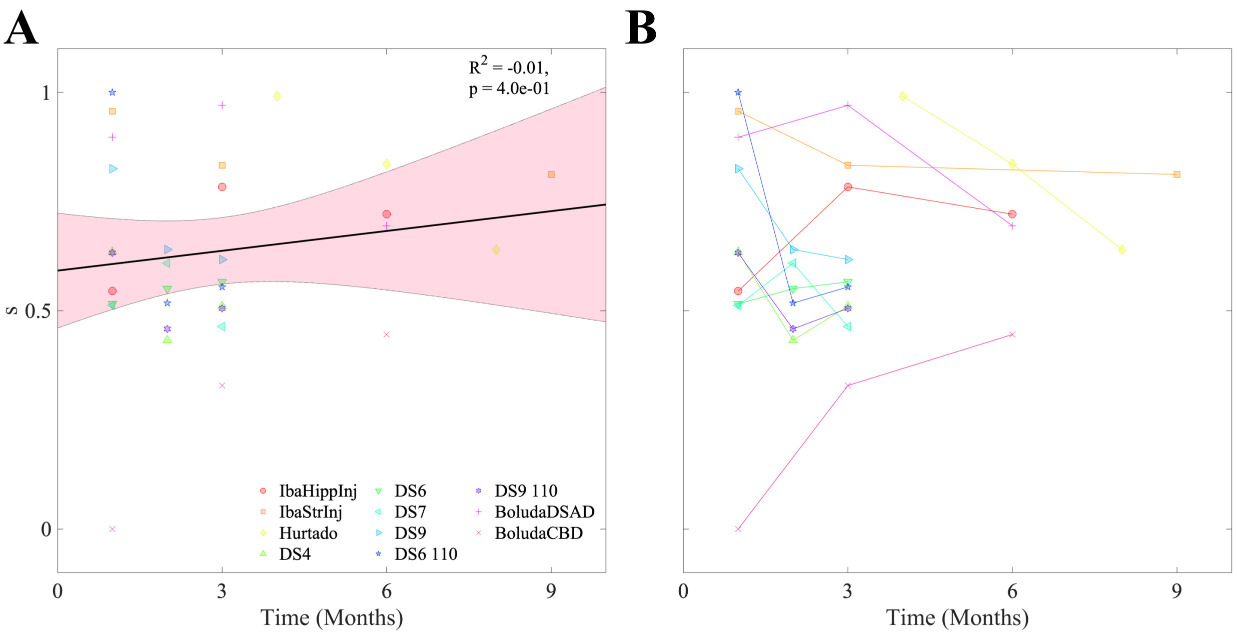

Figure S3: There is no net temporal relationship across studies with respect to directionality bias A. Linear regression reveals that per-timepoint-fit s values do exhibit a shift over time across mouse models. **B.** The same data as **A**, plotted as trajectories for individual mouse models, showing that there is no consistent trend within studies. See **Table S1** for descriptions of these datasets.

References

[1] Iba, M. *et al.* Synthetic Tau Fibrils Mediate Transmission of Neurofibrillary Tangles in a Transgenic Mouse Model of Alzheimer's-Like Tauopathy. *Journal of Neuroscience* **33**, 1024–1037 (2013). URL `https://www.jneurosci.org/lookup/doi/10.1523/JNEUROSCI.2642-12.2013`.

[2] Hurtado, D. E. *et al.* Aβ Accelerates the Spatiotemporal Progression of Tau Pathology and Augments Tau Amyloidosis in an Alzheimer Mouse Model. *The American Journal of Pathology* **177**, 1977–1988 (2010). URL `https://linkinghub.elsevier.com/retrieve/pii/S0002944010602489`.

[3] Kaufman, S. K. *et al.* Tau Prion Strains Dictate Patterns of Cell Pathology, Progression Rate, and Regional Vulnerability In Vivo. *Neuron* **92**, 796–812 (2016). URL `https://linkinghub.elsevier.com/retrieve/pii/S0896627316306973`.

[4] Boluda, S. *et al.* Differential induction and spread of tau pathology in young PS19 tau transgenic mice following intracerebral injections of pathological tau from Alzheimer's disease or corticobasal degeneration brains. *Acta Neuropathologica* **129**, 221–237 (2015). URL `http://link.springer.com/10.1007/s00401-014-1373-0`.
