# Supplementary material for: Directionality bias is necessary to explain spatiotemporal progression of pathology in mouse models of tauopathy
Source: bioRxiv. 2024 Jun 6:2024.06.04.597478. Preprint. [Version 1] doi: 10.1101/2024.06.04.597478 (PMC11185722; doi:10.1101/2024.06.04.597478)
Supplement: Supplementary file 1 [file media-1.pdf]

Supplement to "Directionality bias is necessary to explain spatiotemporal progression of pathology in mouse models of tauopathy"

Justin Torok[1], Christopher Mezias[2], and Ashish Raj[1,*]

[1]University of California, San Francisco, Department of Radiology, San Francisco, CA, 94143, United States
[2]Cold Spring Harbor Laboratory, Department of Neuroscience
[*]Address correspondence to ashish.raj@ucsf.edu

Supplemental Figures

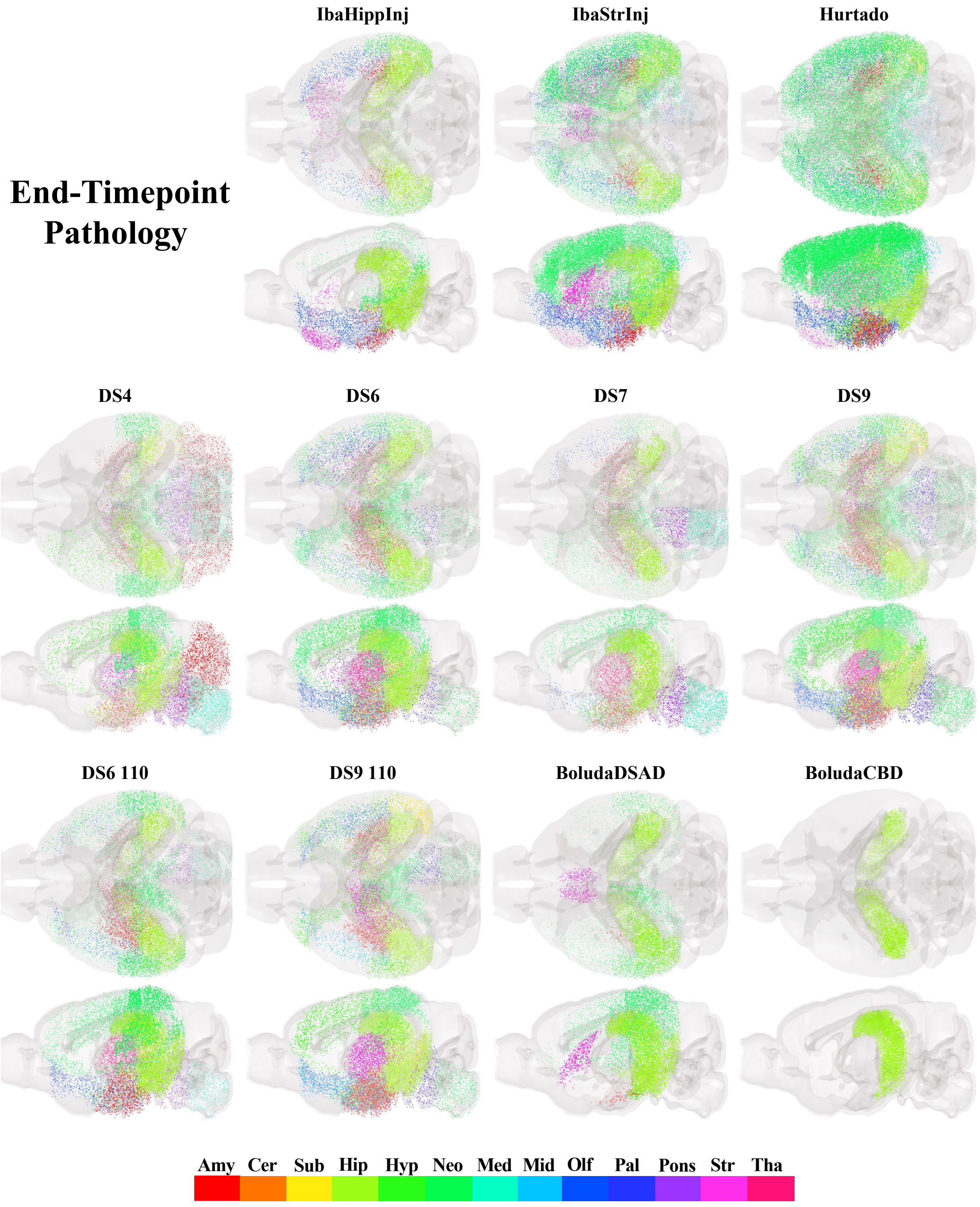

Figure S1: End-timepoint pathology glass brains. End-timepoint pathology for each of the twelve mouse tauopathy datasets, plotted in axial and sagittal views. See **Table 1** for descriptions of these datasets.

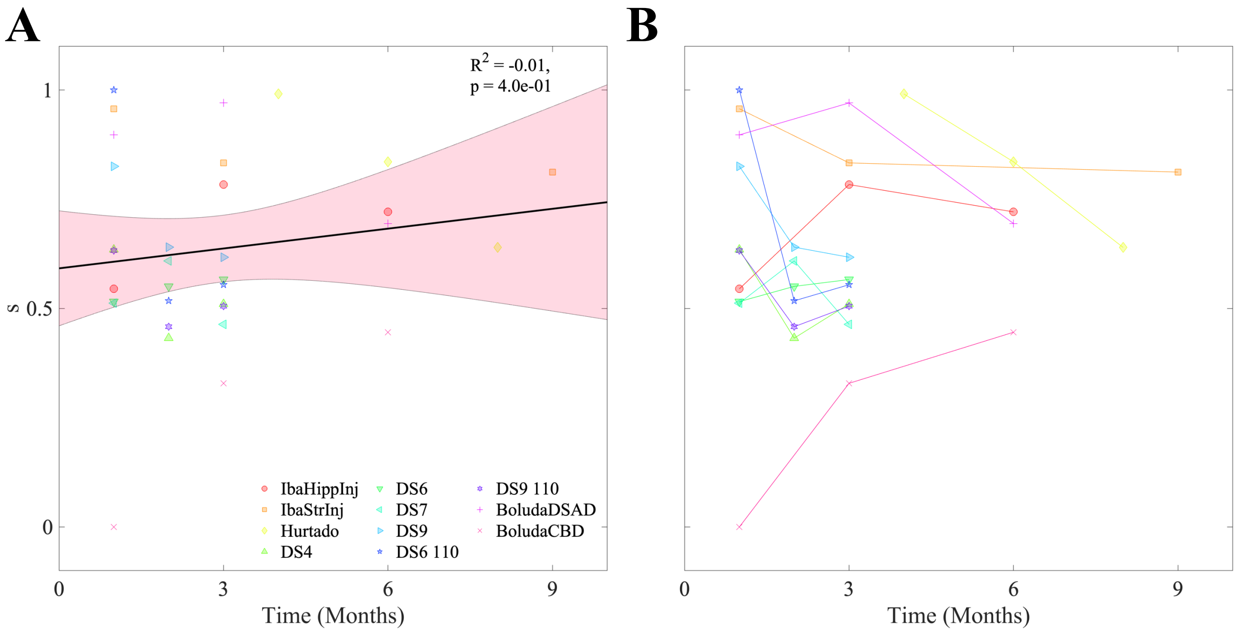

Figure S2: There is no net temporal relationship across studies with respect to directionality bias A. Linear regression reveals that per-timepoint-fit s values do exhibit a shift over time across mouse models. **B.** The same data as **A**, plotted as trajectories for individual mouse models, showing that there is no consistent trend within studies. See **Table 1** for descriptions of these datasets.
